# Urolithiasis in The Pediatric Population − Current Opinion on Epidemiology, Patophysiology, Diagnostic Evaluation and Treatment

**DOI:** 10.34763/devperiodmed.20182202.201208

**Published:** 2018-06-30

**Authors:** Katarzyna Jobs, Magda Rakowska, Aleksandra Paturej

**Affiliations:** 1 Department of Pediatrics, Nephrology and Allergology, Military Institute of Medicine, Warsaw, Poland

**Keywords:** urolithiasis, pediatrics, epidemiology, diagnostics, treatment, kamica, pediatria, epidemiologia, diagnostyka, leczenie

## Abstract

Urolithiasis, a condition in which calculi are found in the urinary tract, has been known for centuries. Although the disease was considered casuistic in the pediatric population, its prevalence is rising among both children and infants. The occurrence of the disease is greater in developed countries, therefore urolithiasis should be considered a lifestyle disease. Its etiopathogenesis has not yet been well understood. Kidney stone formation is influenced by factors such as climate, eating habits, profession, fluid intake, genetic predisposition, urinary tract infections and malformations of the urinary tract. Calculi are usually composed of mixed mineral substances. Only about 30% are made up of one chemical substance, calcium oxalate being the most common. Urolithiasis can be asymptomatic and accidentally diagnosed. Abdominal pain is the most common clinical symptom, however disease presentation among infants is nonspecific. Hematuria is a common clinical finding. Ultrasonography is the most important diagnostic tool in the diagnosis of kidney stone disease. Metabolic evaluation is required in every case of urolithiasis in the pediatric population, as metabolic disorders can be found in the majority of cases in this age group. The spontaneous passage of calculi less than 6mm in diameter is likely. Invasive treatment should be carried out if stones exceed 6mm in diameter or fail to expulse spontaneously. Prophylactic treatment includes adequate fluid intake, healthy eating habits and physical activity to maintain a healthy weight. Urolithiasis is a recurrent disease, therefore long-term treatment, prophylaxis and a lasting change in dietary habits are essential.

## Streszczenie

Kamica układu moczowego, czyli obecność złogów w układzie moczowym, towarzyszy człowiekowi od najdawniejszych czasów. Obecnie zaobserwowano wzrost częstości jej występowania, także u dzieci oraz niemowląt, co uprzednio uważano za kazuistykę. Problem dotyka częściej osób żyjących w krajach wysokorozwiniętych, dlatego kamicę układu moczowego zaczęto nazywać chorobą cywilizacyjną. Etiopatogeneza kamicy nie została dotychczas dokładnie poznana, wiadomo jednak, że na powstawanie złogów ma wpływ wiele czynników między innymi: klimat, zwyczaje żywieniowe, rodzaj wykonywanej przez chorego pracy, ilość spożywanych płynów, uwarunkowania genetyczne, zakażenia układu moczowego, wady budowy nerek i układu moczowego. Większość kamieni ma mieszany skład mineralny, jedynie około 30% zbudowanych jest z jednego związku chemicznego. Najczęściej spotykamy złogi zbudowane ze szczawianu wapnia. Kamica może mieć przebieg bezobjawowy i często bywa wykrywana przypadkowo. Najczęstszym objawem klinicznym jest ból brzucha. U niemowląt objawy mogą być bardzo niespecyficzne. Dość częstą nieprawidłowością wykrywaną w badaniach laboratoryjnych jest krwinkomocz. Najważniejszym badaniem obrazowym w diagnostyce kamicy jest badanie ultrasonograficzne. Każde dziecko z kamicą powinno mieć przeprowadzone dokładne badania dotyczące ewentualnego metabolicznego podłoża choroby ponieważ u większości pacjentów pediatrycznych można wykazać metaboliczne predyspozycje do tworzenia złogów. Większość kamieni o wielkości nie przekraczającej 6 mm w najszerszym wymiarze, może zostać samoistnie wydalona, pozostałe − większe lub po nieudanej próbie leczenia ekspulsywnego, powinny być zakwalifikowane do leczenia zabiegowego. Postępowanie profilaktyczne obejmuje odpowiednią podaż płynów, stosowanie zasad „zdrowego odżywiania”, aktywność fizyczna dla utrzymania prawidłowej masy ciała. Kamica układu moczowego jest chorobą o charakterze nawrotowym, tak więc leczenie, profilaktyka i trwała zmiana zwyczajów żywieniowych powinny obejmować praktycznie całe życie chorego.

## Introduction

Urolithiasis is a condition in which calculi are found in the upper and lower urinary tract. It has accompanied human beings for centuries. Urolithiasis was known already in Ancient Egypt, as seen in the investigations of mummies and on papyri dated 1500 BC. Invasive treatment was known in India as early as in the 6^th^ century BC. Serious complications occurred frequently, therefore conservative treatment with herbs of diuretic and decontaminating properties was attempted. Different age groups were affected, mostly adults [1]. Urolithiasis treatment changed over the centuries; the need for prophylaxis was emphasized and therefore a search for causes of the disease had begun.

Urolithiasis morbidity increased in recent years, hence the greater interest in the disease in recent times [2, 3, 4]. Children and neonates are also affected nowadays, which earlier was considered casuistic.

Many authors suggest that especially in the pediatric population, urolithiasis should be considered more as a symptom than a disease. Therefore, diagnosis must be followed by a thorough investigation of the possible causes, as well as long-term specialist care [1, 3, 4, 5].

## Epidemiology

Urolithiasis is usually considered an adult patients’ disease, however an increasing prevalence of the disease is also being observed in the pediatric population. This is linked to the development of civilization, changes in the diet and lifestyle and possibly also the greater availability of ultrasonography [2, 3, 4]. The disease is usually diagnosed in patients between 20 and 60 years of age [6, 7]. In the literature, the reported incidence of urolithiasis significantly varies between different studies. In German records urolithiasis is estimated to affect 4% of females and 5.5% of males [1, 8]. Other authors suggest that up to 6-7% of women and 11-15% of men in the European population are affected [6, 7]. Approximately 10% of all urolithiasis cases are diagnosed among children [1]. In our latitude, urolithiasis among pediatric patients is estimated to affect 2% of the population. It is worth noting that a fivefold increase of the disease’s occurrence among children has been observed in the last decade [9].

The prevalence of the disease was thought to be greater in the male population. However, no sex or age differences are observed in the last decade’s increase in morbidity [6].

A changing trend in the prevalence rates among children has been observed also in recent years. Previously, urolithiasis was considered to be more common among male pediatric patients, yet American authors report no sex difference and an increasing prevalence of the disease among female adolescent patients [1, 3, 9]. A possible link with the “obesity epidemic” is suggested [2, 3, 6]. The disease is also associated with hypertension and diabetes mellitus [6].

Significant geographical differences are present. Urolithiasis is more likely to occur in the Western Hemisphere, especially the United States of America. Citizens of developed countries, whose diet is rich in animal protein are particularly affected. Therefore, urolithiasis should be considered a lifestyle disease. A close relationship with diet can be observed in Saudi Arabia, where both the intake of animal protein and the disease’s occurrence are the highest in its geographical region [10]. People of Caucasian origin have a greater predisposition to form deposits in the urinary tract [10]. A low prevalence of the disease is observed in the African population, which is worth noting, considering the possible association of the disease with warm climate [9].

A metabolic predisposition to form deposits is found in the majority of pediatric patients, which is a relatively new trend [3, 4, 10, 11]. As showed by British records, the disease’s etiology has changed over the last 30 years from prevalent infectious causes to metabolic reasons [9].

As demonstrated by the records from developed countries, deposits are most commonly located in the renal pelvis and ureter, rarely in the bladder [1, 3, 9].

A high recurrence rate of urolithiasis is observed: up to 80% according to the literature [3, 4, 6, 11]. Unless prophylactic treatment is implemented, new deposits will form in 35% to 50% of all cases [6, 12]. A much higher recurrence rate is observed in the pediatric population than in adults [3]. However, these records are based on out-of-date data and should be carried out again taking into account contemporary data [4].

Familial occurrence of urolithiasis is noted in about 40% of the cases [1].

## Etiology and pathogenesis

The etiopathogenesis of the disease has not yet been well understood. The impact of environmental factors, such as climate, eating habits, profession, fluid intake is suggested [2, 3, 9]. Furthermore, genetic predisposition is reported and certain metabolic disorders are considered. Stone formation is probably caused by the interaction of both factors [1, 3].

No single gene causation has been reported, as urolithiasis can occur in the course of many different pathologies and conditions. Kidney stone disease is likely inherited polygenically. However, rare single-gene disorders, with urolithiasis as one of their symptoms, have been noted. These diseases are usually inherited in an autosomal recessive or X-linked recessive pattern. Examples include primary hyperoxaularia, cystinuria, familial hypomagnesemia with hypercalciuria and nephrocalcinosis (FHHNC), Bartter’s syndrome, type I glycogenosis, xanthinuria, Dent disease, Lowe syndrome, Lesch-Nyhan syndrome and distal tubular acidosis [1, 5]. A link is suggested in recent research between urolithiasis and mutations in genes regulating vitamin D3 (CYP24A1) metabolism and phosphate transport (SLC34A3) [1, 5, 9]. A new mutation has been described recently in the SLC26A1 gene, which may be the first known monogenic hyperoxaluria that is associated with excessive absorption of oxalate from the gastrointestinal tract [13].

Finally, urolithiasis is linked with urinary tract infections, urinary tract malformations leading to urine retention, malnutrition and use of medications [1, 3, 8, 9, 14].

The process of stone formation can be divided into the following phases: nucleation, crystal growth, aggregation and retention. In the nucleation phase, urine is supersaturated with either physiological or pathological substances and subsequently a deposit is formed. In the crystal growth phase, an increase in the volume and size of small crystals is observed, followed by the aggregation of crystals. In the retention phase the urinary passage is blocked by the deposit. Urine tract obstruction is favored by hindered urine flow and the disadvantageous localization of the kidney stone (such as in the lower renal calyx). Therefore, the higher prevalence of kidney stones among patients with urinary tract malformations can be partially explained (such as polycystic kidney, medullary sponge kidney). A rapid increase in the size of the deposit may also result in the obstruction of the urinary tract [8, 9].

Coe and Evan are authors of the theory of stone formation which is comprised of three different pathways [15]. In the first pathway, Randall’s plaque is deposited in the interstitial tissue of the renal papilla [1, 3]. The plaque, seen as white lesions, is found during endoscopic or surgical procedures. It is composed of calcium phosphate (apatite), which accumulates in the basal membrane of the thin segment of Henle’s loop and then migrates to the interstitial space of the renal papilla. If the epithelium covering the plaque is damaged, calcium oxalate crystals build up, leading to stone formation. Calcium oxalate stones are formed in this pathway. Idiopathic hypercalciuria, the most common type of urolithiasis is associated with calcium oxalate deposits. Possibly, a genetic defect of the proximal tubes’ function is related with the disease. In this pathway no signs of inflammation or fibrosis are found within Randall’s plaques and no crystals are found within the renal tubules. Therefore, kidney stones that form in this mechanism do not lead to chronic renal disease [15].

In the second pathway reported by Coe and Evan, crystals are formed in the renal tubules and are accompanied by inflammation, fibrosis and loss of tubules. These processes are caused by an underlying disease that affects the tubules and medullary interstitium and may lead to chronic renal disease. Stones in the course of tubular acidosis are formed using this mechanism. Stones composed of calcium phosphate are also formed via this pathway and are in part attributed to primary hyperparathyroidism [15].

In the third pathway, deposits are formed via free crystallization in the solution, a result of urine supersaturation by a stone-forming substance and its poor solubility. The expulsed deposits show no traces of attachment to the epithelium of the renal pelvis. Renal stones are formed in this mechanism in the course of cystinuria and among patients with ileostomy or colostomy [15].

Urine pH is an important factor in the pathophysiology of stone formation [3, 12]. Uric acid stones are formed in an acidic environment, whereas phosphate deposits are formed in an alkaline environment. The formation of calcium oxalate stones is least influenced by pH, nevertheless pH 6.0-7.0 is considered the least favourable [3, 12].

Stone formation is also influenced by the concentration of crystallization promoters and inhibitors found in urine [3]. Citrates and magnesium are known crystallization inhibitors. Furthermore, zinc, pyrophosphates, glycosaminoglycans (produced by epithelial cells of the renal tubules) are suggested to hinder stone crystallization. Additionally, several proteins are researched for its role in the crystallization process as, depending on the circumstances, stone formation is either promoted or inhibited. The most widely known protein inhibitors of crystallization include: uromodulin (Tamm Horsfall protein), osteopontin (uropontin), osteocalcin (nephrocalcin) and calgranulin [5, 8].

All the substances with a crystallization potential are considered stone-formation promoters. These include calcium, oxalate, cystine, uric acid and other substances which form the stone nucleus: bacteria, foreign bodies, exfoliated epithelial cells [5, 8].

Process of lithogenesis is promoted by decreased urine output, which results in more concentrated urine [3, 5, 8].

Stone formation may also be affected by drugs, such as indinavir, triamterene and ceftriaxone [3, 5].

Kidney stones can be called biominerals, because although they are mainly composed of inorganic substances, they also contain an organic component [16].

Urolithiasis with calcium oxalate deposits (either monohydrate or dihydrate) is the most common type and accounts for 80% of all kidney stone cases in both the adult and pediatric population. Other stones are composed of uric acid, struvite (magnesium ammonium phosphate), calcium phosphate (brushite, hydroxyapatite, carbonate apatite) or cystine. Stones containing xanthine and 2,8-dihydroxyadenine are rare [4, 5, 8].

Most stones have a mixed mineral composition. Only about 30% are made up of one chemical substance. Deposits can be formed of either layers of different minerals or homogeneous mixture of different compounds. The chemical compound that has the highest percentage in composition defines the stone type [8].

The formation of stones made of struvite, ammonium urate or carbonate apatite is linked to urinary tract infections with urease-positive bacteria, such as Proteus sp., Klebsiella sp. and Serratia marcescens. Urea is broken down by these bacteria into ammonia and carbon dioxide. Urine is therefore alkalized by resultant ammonium ions and crystallization is promoted [3, 5, 11].

The most common metabolic disorder in both adult and pediatric patients with urolithiasis is idiopathic hypercalciuria (IH). It is diagnosed when the patient presents with increased urinary calcium excretion and normal blood calcium level. Additionally, there are no abnormalities in other metabolic tests, except hypomagnesuria and hypocitraturia observed in some patients with this disorder. In children increased urinary calcium excretion means over 4 mg per kg of body weight per day. Symptoms of IH are nonspecific, the same as in other forms of urolithiasis. Historically, three forms of hypercalciuria, based on Pak’s test, were distinguished: absorptive, renal and resorptive. Lately, there has been a theory that they are manifestations of the same defect and Pak’s test is seldom used. There are no differences in treating the absorptive and renal form of this abnormality. It is likely that IH is polygenic in nature with varying expression of multiple genes among patients. Patients with IH could have an increased number of vitamin D receptors or show higher susceptibility to it [17].

## Clinical manifestations of urolithiasis in children

Children rarely present with classic symptoms of renal colic and nonspecific symptoms are much more common. Furthermore, urolithiasis can be asymptomatic for a long period of time and can be detected accidentally [1, 3, 5].

The most prevalent symptom is abdominal pain, which younger children do not present in a typical localization. Other symptoms, especially among infants, include anxiety, refusal to eat, poor weight gain and constipation. All these symptoms can result from abdominal pain [1, 3].

Urolithiasis must be considered among patients with recurrent urinary tract infections and sterile pyuria. Microscopic and gross hematuria are characteristic for kidney stone disease and should be followed by diagnostic imaging. Hematuria often precedes the stone formation and is usually explained by abnormal crystalloid excretion [1, 3]. Other symptoms of urolithiasis include dysuria, nycturia, enuresis and urinary urgency [3].

Symptoms among children vary depending on the stone localization. Stones in the lower urinary tract cause dysuria, polyuria, enuresis, nycturia and pain that radiates to the groin. Stones descending from the renal pelvis cause pain in the lumbar region, anxiety, nausea and vomiting. The intensity of pain correlates with the level of urinary blockage [3, 5].

Anuria and acute renal failure occur rarely and may be a result of the complete obstruction of urine flow: blockage of the urethra, ureter of a single kidney or massive bilateral urolithiasis [5].

Calcium oxalate urolithiasis in the course of idiopathic hypercalciuria, i.e. the most common cause of kidney stones, is associated with reduced bone mineral density [14].

## Diagnosis of urolithiasis – Imaging studies

Ultrasonography is the most important and simplest imaging study in the diagnosis of kidney stone disease. It is a highly sensitive test in the hands of a skilled ultrasonographer. However, deposits located in the ureter, especially in its middle section, are difficult or impossible to visualize. A typical stone is seen as a hyperechogenic structure with a posterior acoustic shadow and twinkle artefact in color Doppler ultrasound [1, 3, 5]. Stones up to 2-3 mm in diameter can be visualized with ultrasound [3, 4].

If diagnostic difficulties are encountered, non-contrast spiral computed tomography should be carried out [3, 4]. The availability of the method is increasing and the study is characterized by high sensitivity and specificity. However, it should not be a method of first choice due to radiation. The radiation dose during standard protocol is about 3.7 mSv and 0.5-0.7mSv in low dose protocols [5].

With the development of modern imaging techniques, abdominal X-ray is a diagnostic tool that is rarely used. It can be used to visualize the exact location of the deposit prior to extracorporeal shock wave lithotripsy (ESWL). Additionally, kidney stone composition can be preliminarily assessed in an abdominal X-ray. Calcium phosphate and calcium oxalate stones are radiopaque, struvite and cystine calculi are faintly radiopaque, while uric acid stones are entirely radiolucent. It must be remembered that the radiation dose of a traditional X-ray exam ranges from 0.5 to 0.9 mSv [1, 5].

## Metabolic evaluation of urolithiasis

Various diagnostic algorithms are found in the literature. Nevertheless, all the authors agree that metabolic evaluation is required in every case of urolithiasis in the pediatric population [1, 3, 4, 5]. Metabolic disorders can be found in the majority of cases in this age group [3, 5].

When starting evaluation, the patients’ medical history should be thoroughly investigated and particular attention should be paid to previous bone fractures and immobilization periods. Points of interest include episodes of hematuria, abdominal pain, urinary tract infections and sterile pyuria, family history of urolithiasis and other diseases. Furthermore, special attention should be drawn to the patients’ diet, fluid intake and use of medication [1, 6].

Metabolic evaluation should be carried out in the time period after stone expulsion and should be repeated several times. Evaluation includes blood tests, a second morning urine sample and a 24-hour urine collection. Proper analysis is possible if usual diet and normal lifestyle is maintained and after excluding urinary tract infection [1, 3, 6, 8, 11].

Laboratory evaluation is usually divided into two stages: preliminary and thorough. Preliminary tests allow the precise planning of the subsequent tests. Furthermore, these tests ensure that further studies will yield interpretable results. Interpretable data may not always be obtained, for example among patients with chronic kidney disease.

At the preliminary stage, urinalysis should be carried out, special attention should be drawn to specific gravity, pH, leukocyte esterase, the presence of white, red blood cells, electrolytes. Urine culture should be carried out to exclude urinary tract infection (interpreted together with leukocyturia) [3, 8]. The concentration of creatinine, sodium, potassium, chlorine, calcium, phosphorus, uric acid and blood gases should be analyzed [3, 8].

In the course of thorough evaluation, urinary excretion of creatinine, calcium, uric acid, sodium, phosphorus, magnesium should be evaluated. Oxalate, citrate and cystine urinary excretion should be analyzed if clinical and/ or laboratory indications exist [3, 8]. These are tested for in both 24-hour urine analysis collection and in the second morning urine sample. Excretion of these ions in the second morning sample should be related to the excretion of creatinine [11]. The tests should be repeated to acquire accurate test results. Patients should maintain their usual diet [3, 8, 11]. Blood analysis should include the concentration of uric acid, creatinine, sodium, potassium, calcium, phosphorus, magnesium, uric acid and metabolites of vitamin D (25OHD) [14]. If additional clinical and/ or laboratory indications exist, concentration of parathyroid hormone and TSH should be analyzed [1, 3].

All-day pH analysis is a valuable diagnostic aid [3, 8, 12].

It is important that the composition of the expulsed stone is known. However, analysis may be misleading at times and cannot substitute detailed patient evaluation. Currently only three methods are used to evaluate the stone composition: infrared spectroscopy, X-ray diffraction and polarization microscopy [1, 11, 12, 14]. Unfortunately none of these are widely available in Poland.

Metabolic evaluation provides the opportunity to establish prophylactic treatment among all the patients with urolithiasis and makes it possible to implement appropriate conservative treatment. The most important data about excretions in urine of substances important in the diagnostics of urolithiasis in the pediatric population are listed in Table I.

**Table I:** Normal values for 24-hour urine and second fasting morning urine sample [8]. Tabela I. Normy wydalania danych substancji w dobowej zbiórce moczu oraz w drugiej po nocy porcji moczu pobieranej na czczo [8].

	24 h urine *Dobowa zbiórka moczu*	Second morning urine samplefor mg/mmol of creatinine *Druga po nocy porcja moczu na czczo w przeliczeniu na mg/mmol kreatyniny*		
calcium *wapń*	<4 mg (0.1 mmol)/kg	<1 y <1 r.ż. 1-3 y 1-3 r.ż. 3-5 y 3-5 r.ż. 5-7 y 5-7 r.ż. >7 y >7 r.ż.	mg/mg <0.81 <0.53 <0.39 <0.28 <0.21	mol/mol <2 <1.5 <1.1 <0.8 <0.6
oxalate *szczawiany*	<45 mg (0.5 mmol)/1.73 m^2^	<6 mth <6 m.ż. 7-24 mth 7-24m.ż. 2-5 y 2-5 r.ż. 5-14 y 5-14 r.ż. >16 y >16 r.ż.	mg/g <188-260 <110-139 <80 <60-65 <32	mmol/mol <325-360 <132-174 <100 <70-82 < 40
citrate *cytryniany*	>365 mg (1.9 mmol)/1.73m^2^ >310 mg (1.6 mmol) /1.73m^2^	<5 y <5 r.ż. >5 y >5 r.ż.	g/g >0.42 >0.25	mol/mol >0.25 >0.15
uric *kwas* acid *moczowy*	<0.56 mg/dl (33 μmol/l)/GFR			
magnesium *magnez*	>0.8 mg (0.04 mmol)/kg		mg/mg >0.13	mol/mol >0.63
cystine *cystyna*	<10y <13 mg (55μmol)/1.73m^2^ <10r.ż <13 mg (55 μmol)/1.73m^2^ >10 y <48 mg (200 μmol) >10 r.ż <48 mg (200 μmol) Adults <60 mg (250 μmol) Dorośli <60 mg (250 μmol)			
phosphate *fosforany*	TRP 85-95%			

TRP − tubular phosphate reabsorption ratio (TRP=[1-(P u/P bx Creat b/Creat u)]x 100%/*współczynnik cewkowej reabsorpcji fosforanów* (TRP={1-(P m/P s x kreat s/kreat m)]x 100%, P u − phosphate in urine/*P m − fosforany w moczu*, P b– phosphate in blood/*P s − fosforany we krwi*, Creat u − creatinine in urine/*kreat m − kreatynina w moczu*, Creat b − creatinine in blood/*kreat s − kreatynina we krwi*.

## General principles of prophylactic treatment in urolithiasis

Urolithiasis is usually diagnosed with the first deposit located in the urinary tract. At this moment, the method of stone removal should be chosen. Most calculi less than 6 mm in diameter pass spontaneously. Invasive treatment should be carried out if the stones exceed 6 mm in diameter or fail to expulse spontaneously. Invasive methods include endoscopic or percutaneous lithotripsy and the rarely performed, classic surgical treatment [3].

Due to the recurrent nature of the disease, introduction of prophylactic treatment after kidney stones removal is essential. No reliable indicator is known to predict the risk of new stone formation and the activity of the disease cannot be assessed. Therefore, prophylactic treatment is necessary for all the children diagnosed with urolithiasis [3].

Even though kidney stones formation is a result of many disorders and metabolic abnormalities, the general principles of prophylaxis are common for all types of urolithiasis.

The most important recommendation in prophylaxis is adequate fluid intake [1, 2, 9, 11, 12, 14]. Fluid intake should be spread evenly throughout the day and in some cases should be increased before the night sleep. It is believed that drinking fluid is more important than the type of the fluid consumed. However, the intake of sweetened or mineral-rich fluids is not recommended. Orange juice is considered beneficial, whereas apple, cranberry and grapefruit juice should be avoided because they have a greater impact on urine pH [2, 14]. Additionally, low-mineral water (mineral content not exceeding 500mg per liter) should be recommended. Filtered water or boiled tap water can also be advised. The intake of fluid should be adjusted to the specific gravity of the first morning urine sample (reference range 1010-1015). Alternatively, 1.5-2 liters per 1.73 m^2^ of body surface can be advised [8].

Dietary recommendations practically overlap with healthy lifestyle principles. The diet should be varied and well-balanced with vegetables, whole grain products and plant lipids [2, 3, 11]. Calcium intake must not be restricted, the diet should contain between 800 and 1200 mg calcium per day. Adequate calcium intake is essential, as calcium ions are partially bonded with oxalates in the gastrointestinal tract and hence secondary hyperoxaluria is prevented [1, 2, 3]. Saline supply should be limited and not exceed 100 mmol per day. Sodium urinary excretion should not exceed 130 mmol/l [2, 3, 6]. The animal protein intake should be limited to 0.8-1.0 g/ kg. Higher animal protein intake than recommended increases calciuria, reduces citrate excretion and promotes the crystallization of calcium oxalate on the crystals of sodium urate [3]. Finally, sweets and dietary supplements (vitamins, minerals) should be avoided [9, 12,14].

General lifestyle recommendations include maintaining an adequate body weight (BMI of 18-25 kg / m2), which should be a result of both diet and adequate physical activity. In addition, proper stress management, adequate sleep and avoidance of excessive fluid loss are recommended [3, 8, 11].

## Conservative treatment of urolithiasis

Long-term medical care of a patient, should comprise of prophylaxis and pharmacotherapy specific for the type of kidney stones.

In the treatment of calcium oxalate urolithiasis with excessive oxalate secretion, oxalate-rich products should be avoided (rhubarb, sorrel, spinach, cocoa, tea or cola type drinks) [2, 3, 5, 8]. A normocalcemic diet should be maintained in order to reduce the oxalate absorption in the gastrointestinal tract. If the condition is accompanied by hypocitraturia, supplementation of potassium citrate or magnesium citrate with the addition of vitamin B6 is advised [1, 2, 3, 6]. If the condition presents with severe clinical symptoms (hematuria, abdominal pain, high rate of stone formation) or is accompanied by reduced bone mineral density, administration of hydrochlorothiazide is recommended in order to decrease calcium excretion [1, 2, 4, 9, 11]. Guidelines for vitamin D daily supplementation have recently changed [14]. Studies show that relatively high vitamin D doses do not cause or aggravate hypercalciuria. On the other hand, adequate intake of vitamin D reduces the crystallization of calcium oxalate in the urinary tract [14]. However, these findings do not regard children, especially infants, with hypersensitivity to vitamin D or its overdose. These patients usually present with hypercalciuria, hypercalcemia and increased concentration of 25OHD3 [18]. In the rest of the patients vitamin D supplementation should be recommended in individually selected doses. It is necessary to check calcium excretion in the urine every three months.

Hyperuricosuria and acidic urine favors the formation of uric acid stones. Conservative treatment is usually the method of choice, as alkalization of urine leads to dissolution of the deposits. Treatment of uric acid stones should be accompanied by increased fluid intake and urine pH in the range of 7.0-7.2. In the prophylaxis of uric acid stone formation, a pH of 6.5-6.8 is recommended. A vegetable and fruit (especially citrus) rich diet is recommended. At times, the administration of potassium citrate is advised. The intake of animal protein should be reduced and purine-rich foods should be limited (organ meats, broth, legume seeds). A limited supply of saline is advised. Supplementation of substances that acidify urine is contraindicated (vitamin C, cranberry). If hyperuricemia is present, allopurinol is administered [1, 2, 3, 4, 5].

Struvite (magnesium ammonium phosphate) stone formation is a result of urinary tract infections with urease-positive bacteria. Expulsion of all the deposits is essential, as even small leftover fragments predispose to recurrent urinary tract infection and new stone formation. Antibacterial treatment is recommended. Other useful methods include urine acidification, limited intake of phosphate-rich foods and magnesium supplements [1, 2, 11].

Cystine kidney stones form in the course of cystinuria, which is a genetically conditioned tubulopathy. Increased fluid uptake (greater than 1.5l/m^2^ among children) is essential in the treatment. Furthermore, the intake of saline and methionine (meat products, eggs) should be limited, especially in the evening hours. Increased solubility of kidney stones is obtained by alkalization of urine above pH 7 with the use of potassium citrate. If such treatment proves insufficient, pharmacologic treatment should be administered. D-penicillamine and tiopronin form soluble compounds with cystine, which are relatively easily excreted by the kidneys. Unfortunately, such treatment is accompanied by several side effects. Captopril is also used, especially among patients with hypertension or post-inflammatory nephropathy. However, the effectiveness of this treatment is still questioned [1, 2, 3, 6, 9, 11].

Apart from long-term prophylactic treatment, all deposits should be expulsed. This is particularly important among patients with renal colic. Pain is a result of increased pressure in the renal pelvis caused by the kidney stone blocking the urinary tract. This condition stimulates prostaglandin production, which causes urethra contractions, dilation of the renal vessels, increased diuresis and irritation of the nerve fibers [3]. Painkillers and drugs facilitating the stone expulsion should be administered, and such treatment is called medical expulsion therapy (MET) [4, 19].

Nonsteroidal anti-inflammatory drugs (NSAIDs) are most commonly used in analgesic treatment. Opioids are administered in case of persistent, unbearable pain, and can be used alongside NSAIDs [3]. According to some authors, desmopressin can also be a useful addition to NSAIDs, due to its antidiuretic effect and inhibition of renal pelvis contractions. Careful monitoring of fluid balance is required to prevent overhydration [3]. However, the recommendation of desmopressin is controversial.

NSAIDs enhance stone expulsion, as they reduce the edema caused by inflammation. However, they do not influence the speed with which stones are expulsed. Other commonly used drugs are α-blockers (tamsulosin, doxazosin, alfuzosin), which facilitate the expulsion of deposits located in the ureter [3, 4, 19, 20], tamsulosin being the most effective. Descriptions of nifedipine use (calcium channel blockers) are found in the literature. Nifedipine blocks spontaneous activity of the urethra and calyces, nevertheless it is less effective than tamsulosin [3]. Suggestions are found in the literature for corticosteroid use in the expulsion therapy, where their anti-inflammatory effect can facilitate the deposit expulsion. Their use with a-blockers or nifedipine seems beneficial, however, corticosteroids should not be administered in monotherapy [3, 19].

Aggressive hydration is not recommended, as it can aggravate pain and lead to obstruction of the urinary tract by the deposits [3, 5, 19].

## Invasive treatment of urolithiasis

Up to 80% of urinary deposits can be expulsed spontaneously. The least invasive procedures should be performed in order to remove the remaining kidney stones. These methods include extracorporeal shock wave lithotripsy (ESWL), lithotripsy during ureteroscopy (URSL), percutaneous nefrolithotripsy (PCNL) and retrograde intrarenal surgery (RIRS) [3]. Classic surgical treatment is necessary only in a few selected cases. Urinary tract malformations accompanying urolithiasis are one of the indications for open surgery, as such treatment allows the simultaneous correction of the anatomical abnormalities.

## Prognosis

Urolithiasis is a recurrent disease, therefore long-term treatment, prophylaxis, repeated control metabolic tests and a lasting change in dietary habits are essential. No reliable indicator is known to predict the risk of new stone formation, therefore the activity of the disease cannot be assessed. Nevertheless, observatory studies indicate that 80% of the patients relapse if left untreated (between 50 and 100% depending on the deposit type). The recurrence rate among the patients treated is 10-15% [3, 12]. It is also known that familial occurrence of urolithiasis is noted in about 40% of the cases [1, 3].

Prognosis is also dependent on the type of urolithiasis [14]. It is very important to note that chronic kidney disease does not develop in the course of idiopathic hypercalciuria. Patients with recurrent urinary tract infections and obstruction of urinary tract present with worse prognosis [14]. Therefore, prognosis is particularly influenced by adequate treatment.

## Summary

Urolithiasis is actually considered a systemic disorder associated with such abnormalities as diabetes, obesity and hypertension. Although in children the link between urolithiasis and metabolic syndrome is weak, we should take into consideration the obesity epidemic in young patients and plan future scientific research in this aspect [21].

Recent epidemiologic data in adults indicate the connection between urolithiasis and chronic kidney disease. It is less clear in the population of children but, as mentioned above, it depends on the type of disease leading to urinary stones [21].

All the children with urolithiasis should have metabolic diagnostics and remain under medical care. It is important to repeat metabolic tests at least once a year, as they could show a different pattern with time.
